# Effect of sex-specific abdominal fat tissue composition on WHO/ISUP nuclear grade of clear cell renal cell carcinoma

**DOI:** 10.55730/1300-0144.5850

**Published:** 2024-03-04

**Authors:** Eser BULUT, Ali KÜPELİ, Mehmet Akif RAMAZANOĞLU, Hasan Rıza AYDIN, İbrahim SİBAL, Fatih BIÇAKLIOĞLU, Fatih YILDIRIM, Şenol ADANUR, Salih AL

**Affiliations:** 1 Department of Radiology, Faculty of Medicine, Trabzon Kanuni Training and Research Hospital, Trabzon, Turkiye; 2 Department of Urology, Faculty of Medicine, Trabzon Kanuni Training and Research Hospital, Trabzon, Turkiye; 3 Department of Urology, Faculty of Medicine, Atatürk University, Erzurum, Turkiye

**Keywords:** Clear cell carcinoma, renal cell carcinoma, obesity, computed tomography, neoplasm grading

## Abstract

**Background/aim:**

To investigate the relationship between sex-related visceral obesity and WHO/ISUP nuclear grade in clear cell renal cell carcinoma (ccRCC).

**Materials and methods:**

Between January 2018 and June 2022, 95 patients (56 men and 39 women) with pathologically proven ccRCC who underwent abdominal computed tomography examination were retrospectively examined. The patients were classified into two groups: low- and high-WHO/ISUP nuclear grade ccRCC (n = 58 and n = 37), respectively. Patient height, weight, body mass index (BMI), sex, age, subcutaneous fat area (SFA), visceral fat area (VFA), total fat area (TFA), and percentage of visceral fat (VF%) were recorded for the two groups.

**Results:**

No significant differences were found in age, BMI, SFA, or TFA, but VFA and VF% were significantly higher in the high-grade patient group. In males, maximal tumor diameter (MTD) (67.8% sensitivity and 76.9% specificity) had the highest area under the curve (AUC), while in females, VF% (70.0% sensitivity and 73.7% specificity) had the highest AUC. VF% revealed an odds ratio (OR) of 1.09 in females with high-grade ccRCC, and in males, MTD was an independent predictor of ccRCC with an OR of 1.03.

**Conclusions:**

Sex-related body fat tissue, including VFA and VF%, could be used for estimating WHO/ISUP nuclear grade in patients with ccRCC, especially in females.

## 1. Introduction

Renal cell carcinoma (RCC) is the most common primary malignant tumor of the kidney in adults and accounts for approximately 90%–95% of renal tumors [1]. Among the subtypes of RCC, clear cell renal cell carcinoma (ccRCC) is the predominant subtype, and the biological aggressiveness of ccRCC significantly changes the prognosis [2]. Numerous histopathological features, such as TNM stage, tumor size, tumor grade, coagulative necrosis, and microvascular invasion, have been determined to affect the postoperative prognosis of patients with ccRCC [3]. Among these prognostic determinants, nuclear grading of carcinoma is widely known as an important independent factor for cancer-specific survival in ccRCC patients [4].

The World Health Organization/International Society of Urological Pathology (WHO/ISUP) grading system for ccRCC has increased the interobserver reproducibility. Additionally, it is more clinically relevant and easier to apply than the former Fuhrman grading system [5]. In the WHO/ISUP grading system, grades 1–3 are defined based on nucleolar prominence, and grade 4 is determined by the presence of highly atypical nuclear pleomorphism, sarcomatoid, or rhabdoid morphology differentiation [6]. While grades 1–2 are described as low grades, grades 3–4 are defined as high grades.

Obesity is described as an excess of body fat. Clinically, it is usually evaluated with an increase in body weight and body mass index (BMI), but obesity is quite a heterogeneous condition [7]. Visceral obesity has been shown to be strongly related to different malignant tumors, such as esophageal cancer, breast cancer, pancreatic cancer, lymph node metastases, and RCC [8–11]. It has been shown that visceral obesity is closely connected with ccRCC [12]. Since the distribution of body fat is different by sex, men are prone to have more visceral fat tissue, and women are prone to have more subcutaneous fat tissue [13]. Nguyen et al. reported that sex differences in visceral fat tissue can affect the overall survival in patients with RCC, and Hu et al. showed that females can have a higher nuclear grade related to increased visceral fat tissue than males [14,15].

The visceral fat tissue can be exactly evaluated and measured with presurgical computed tomography (CT) scans to determine visceral obesity [16]. However, there are only a few studies on the relationship between sex-specific visceral fat tissue and WHO/ISUP nuclear grade of ccRCC. In this study, we aimed to investigate the association between sex-specific abdominal fat tissue composition according to CT and the WHO/ISUP nuclear grade of ccRCC.

## 2. Materials and methods

### 2.1. Study population

This retrospective study was approved by the Institutional Ethics Committee and complied with the Declaration of Helsinki. Data from January 2018 to June 2022 were obtained through an electronic search of the Picture Archiving and Communication System (PACS). The inclusion criteria were as follows: available presurgical CT scans, pathologically proven ccRCC, and WHO/ISUP nuclear grades. The exclusion criteria were prominent artifacts on CT images due to motion or metal, ccRCC without WHO/ISUP nuclear grades, lack of fit on axial abdominal CT images, and recent significant weight changes. Finally, based on the histopathologic analyses, 95 patients with ccRCC were included in the study. Demographic data, including the patient’s age, sex, height (m), and body weight (kg), were noted for the patient from the medical files. Additionally, BMI (kg/m^2^) was calculated as the ratio of total body weight to height squared for patients in both groups.

### 2.2. CT protocol

Abdominal CT examinations were performed with the participants in the supine position, and the patients were scanned from the diaphragm to the pubic symphysis using a 128-slice CT scanner (GE Optima CT660, GE Healthcare, Milwaukee, WI, USA). All patients were injected with a total of 100–120 mL of nonionic contrast agent and 30 mL of saline at a flow rate of 4 mL/s via the antecubital vein with mechanical power injectors according to the portal venous phase with a start delay of 70 s. The CT protocol was as follows: 120 kVp, tube current of 150–165 mAs, maximum collimation of 2.5 mm, slice thickness of 1.5 mm and rotation time of 0.5 s. The images were then reconstructed into multiplanar reformations.

### 2.3. Image analysis

**Figure 1:**
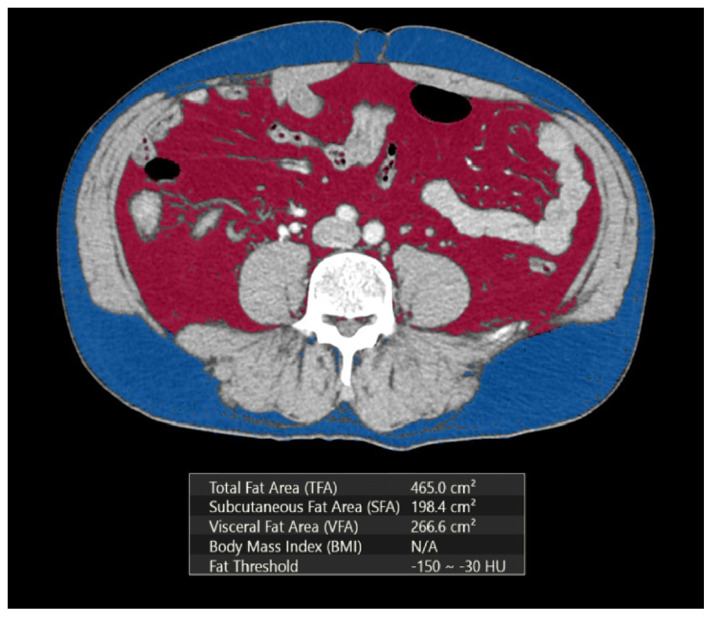
Automatic calculation of visceral and subcutaneous fatty tissue in the range of −150 and −30 HU on the CT image in the axial plane at the level of the umbilicus.

All CT examinations were reevaluated by two radiologists (E.B. and A.K., with 8 and 10 years of abdominal radiology experience, respectively) to reach a consensus without knowing the WHO/ISUP nuclear grades of the lesions and demographic data. The CT images were transferred to a workstation for evaluation. The maximum tumor diameters (MTD) were measured at the axial slice. Next, the cross-sectional abdominal visceral (VFA) and subcutaneous fat areas (SFA) were measured from CT images at the umbilical level. The VFA, TFA, and SFA values were obtained by setting the attenuation values for a region of interest between −150 and −30 HU according to a previous study (Figure 1) [16]. While the VFA was determined as fat tissue between the transversus abdominis fascia and organ surfaces, the SFA was determined in the area between the abdominal fascia and the dermis. Moreover, the percentage of visceral fat (VF%) was calculated using the following formula: VF% = VFA/TFA × 100.

### 2.4. Histopathological assessment of nuclear grade

The histopathology reports were used to evaluate the WHO/ISUP nuclear grades. A total of 22 patients underwent partial nephrectomy, 14 patients underwent total nephrectomy, and 59 patients underwent radical nephrectomy. All tumors were separated into two groups: low-grade ccRCC (WHO/ISUP grades 1, 2) and high-grade ccRCC (WHO/ISUP grades 3, 4).

### 2.5. Statistical analysis

All the data were analyzed using the Statistical Package for the Social Sciences (SPSS 13.0 Statistical Software, SPSS Inc., Chicago, IL, USA) and the MedCalc Statistical Software package version 16.8 (MedCalc Software Bvba, Ostend, Belgium). Descriptive statistics, including the means and ranges, were calculated for age, height, BMI, MTD, SFA, VFA, TFA, and VF%. The Kolmogorov–Smirnov test was used to identify deviations from a normal distribution, and appropriate tests were selected accordingly. Student’s t-test was used to compare continuous variables. Moreover, the diagnostic performance indexes, including sensitivity, specificity, positive predictive value (PPV), and negative predictive value (NPV), were calculated for these parameters regarding differentiation of low-grade and high-grade ccRCC. Multivariate regression analysis was conducted to elucidate the independent influencing factors affecting the accuracy of nuclear grading. A p-value less than 0.05 was considered statistically significant for all analyses.

## 3. Results

In this study, we analyzed the data of 95 patients (56 male, 39 female) with ccRCC. The characteristics of the patients and their results are presented in Table 1. Tumors were observed in the right kidney in 46 patients and in the left kidney in 49 patients. Fifty-eight of 95 ccRCCs were low-grade, and 37 were high-grade. The mean age, height (m), and weight (kg) of the patients with low- and high-grade ccRCC were 59.1 ± 11 y, 1.59 ± 0.1 m, and 76.3 ± 13.4 kg and 60.3 ± 12 y, 1.60 ± 0.1 m, and 76.0 ± 8.8 kg, respectively. Additionally, the mean BMI (kg/m^2^) values of the patients with low- and high-grade ccRCC were 29.9 ± 6.5 and 28.2 ± 5.3, respectively. No statistically significant differences were found in mean height, body weight, or BMI between the two groups (p > 0.186).

**Figure 2:**
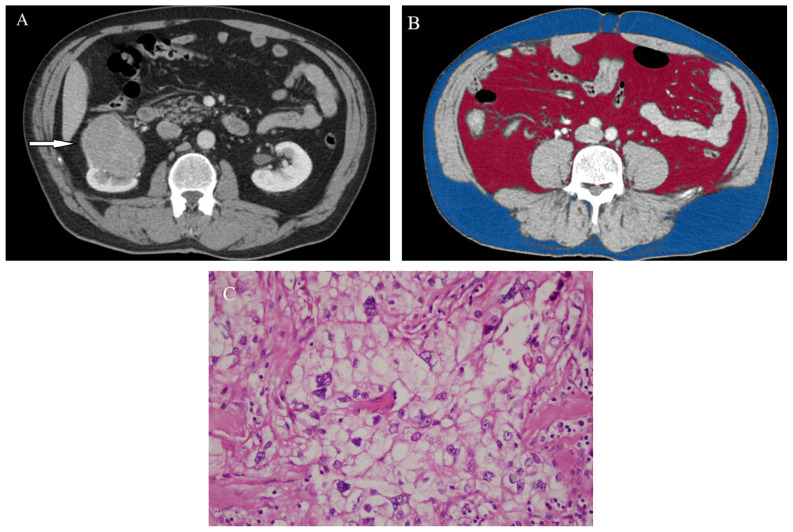
44-year-old man with right-sided clear cell renal cell carcinoma. Contrast enhanced CT (a) shows a tumor (arrow) in the right kidney with a VF% of 57.2% (b). Histologic photomicrograph confirms WHO/ISUP nuclear grade IV ccRCC on high-power (400×; hematoxylin-eosin stain) magnification (c).

**Figure 3:**
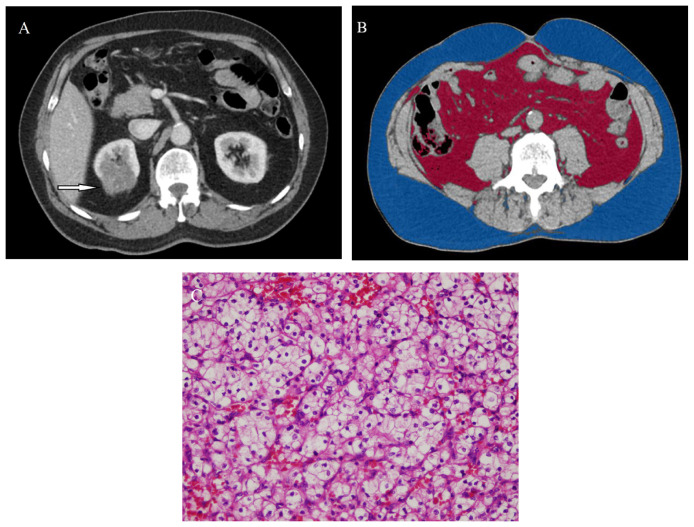
49-year-old man with right-sided clear cell renal cell carcinoma. Contrast enhanced CT (a) shows a tumor (arrow) in the right kidney with a VF% of 39.2% (b). Histologic photomicrograph confirms WHO/ISUP nuclear grade I ccRCC on high-power (400×; hematoxylin-eosin stain) magnification (c).

The mean SFA, VFA, TFA, and VF% values in the patients with low- and high-grade ccRCC were as follows: SFA, 216.0 cm^2^ and 236.1 cm^2^; VFA, 139.5 cm^2^ and 171.6 cm^2^; TFA, 359.3 cm^2^ and 407.8 cm^2^; and VF%, 38.8 and 43.9, respectively. No significant differences were detected in the SFA and TFA values (p > 0.087). While patients with high-grade ccRCC had higher VF% and VFA (Figures 2a–2c), patients with low-grade ccRCC had lower VF% and VFA (Figures 3a–3c). Significant differences were observed in VFA and VF% between the two groups (p < 0.037).

The characteristics of the low-grade and high-grade groups based on sex are shown in Table 2. In males, there was a significant difference in only maximal tumor diameter (MTD) between the low-grade and high-grade groups. In females, the VFA, VF%, and MTD were significantly higher in the high-grade group than in the low-grade ccRCC group.

The ROC curves for VFA, VF%, and MTD are presented in Figure 4. The AUCs were 0.643, 0.627, and 0.735 for VFA, VF%, and MTD, respectively. From the ROC analysis, the optimal cutoff values that provided the highest sensitivity and specificity for VFA, VF%, and MTD were 154.8 cm^2^, 40.7 cm^2^, and 54.0 mm, respectively. The highest diagnostic values acquired for the MTD were 69.4% sensitivity and 65.5% specificity. Using these cutoff values, the diagnostic performance indexes based on sex are shown in Table 3. No significant difference was observed between the AUCs of VFA, VF%, and MTD (p>0.186). In males, MTD (67.8% sensitivity and 76.9% specificity) had the highest AUC, while in females, VF% (70.0% sensitivity and 73.7% specificity) had the highest AUC.

The logistic regression results in the univariate model for determining the associations between variables and WHO/ISUP nuclear grades of ccRCC are shown in Table 4. In the univariate model, VFA (OR, 1.00; 95% CI, 1.00–1.01: p = 0.026), MTD (OR, 1.03; 95% CI, 1.01–1.04: p < 0.001), and VF% (OR, 1.03; 95% CI, 1.00–1.07; p = 0.039) were associated with high-grade ccRCC. Additionally, the logistic regression results of the multivariate model are shown in Table 5. In the multivariate model, VFA (OR, 0.98; 95% CI, 0.97–0.99; p = 0.004) and MTD (OR, 0.95; 95% CI, 0.94–0.97; p < 0.001) were significant predictors of high-grade ccRCC.

## 4. Discussion

Obesity is defined as abnormal or excessive fat accumulation on the body, and BMI is a preferred tool to determine the prevalence of obesity in the population, but obesity is quite a heterogeneous condition [7]. Furthermore, waist circumference measurement is superior to BMI in determining visceral obesity [17]. Abdominal fat distribution, including VFA and SFA, can be precisely measured using MRI or CT to determine abdominal obesity. As an active hormonal tissue, visceral fat tissue releases inflammatory cytokines, insulin, and insulin-like growth factor. These agents can drive the proliferation of cancer cells [18]. It has been reported that visceral obesity carries a risk of malignant tumor development, including tumors such as esophageal cancer, breast cancer, pancreatic cancer, lymph node metastases, and RCC [8–11].

It has been shown that visceral obesity is a significant predictor of survival in metastatic RCC and has a high impact on survival, prognosis, subtype, and grade of RCC [18–20]. Additionally, it has been reported that VFA is an independent prognostic factor for ccRCC, high-grade RCC, and recurrence-free survival in ccRCC [11,12,20]. In contrast, Martini et al. showed that higher total fat was related to survival in metastatic RCC, and Maurits et al. reported that worse overall survival was detected in stage IV RCC patients with lower amounts of visceral adipose tissue [21,22]. Clear cell renal cell carcinoma is the most common and most lethal RCC variant [2]. BMI seems to be an independent predictor of clear-cell histology in RCC. Additionally, the odds of having ccRCC can increase with increased BMI [12].

Although there are several pathological prognostic factors, including nuclear grade, tumor staging, lymphovascular invasion and necrosis, one of the most common prognostic parameters arises from the nuclear grading system for detecting the aggressiveness of ccRCC [3–4]. WHO/ISUP nuclear grading focuses on nucleolar prominence, which is a component of the Fuhrman grading. More objective and simple criteria are used in WHO/ISUP grades 1–3 than in the Fuhrman grading system, which assigns the same importance to nucleolar prominence, nuclear size, and nuclear shape [23]. Needle biopsies are commonly used to diagnose ccRCC; however, they have some limitations in determining nuclear grading, especially in low-grade cases and large heterogeneous tumors [24]. Therefore, there is a need for the accurate prediction of nuclear grade without radical procedures. In this study, we investigated the effect of visceral obesity on predicting WHO/ISUP nuclear grade.

We found that there were no statistically significant differences in terms of height, body weight, BMI, TFA or SFA between the low- and high-grade groups, whereas VFA and VFA% were significantly higher in patients with high-grade ccRCC. Additionally, the MTD was significantly greater in high-grade ccRCC patients than in low-grade patients. In males, the MTD was the only parameter that was significantly higher in the high-grade group than in the low-grade group. In females, the MTD, VFA, and VF% were significantly higher in the high-grade group than in the low-grade group. Hu et al. reported that any of the variables showed a significant difference between the low-grade and high-grade groups in males, and the VFA and VF% revealed significantly greater degrees of elevation in the high-grade group than in the low-grade group in females [15]. While Hu et al. used the Fuhrman nuclear grading system in the pathological grading of ccRCC in their multicenter study, we used the WHO/ISUP nuclear grading system which is a new classification system in our single center study [15].

In our study, the highest diagnostic values provided by MTD showed 69.4% sensitivity and 65.5% specificity. Although the MTD exhibited the highest AUC among the examined variables across both groups, no significant difference was observed between the AUCs. Additionally, no significant difference was found between the AUCs of MTD, VFA, and VF% in the male and female groups. In males, the highest diagnostic values provided by MTD showed 68.7% sensitivity and 76.9% specificity. In females, the highest diagnostic values provided by VF% showed 70.0% sensitivity and 79.7% specificity.

In this study, we separated the patients according to their sex and then classified the lesions into subgroups by nuclear grade to prevent the effect of sex when considering the true effect of visceral fat tissue in predicting prognosis in ccRCC. Although there was no significant difference among VFA and VF% in males, the VFA and VF% were significantly higher in females between low and high nuclear grades of ccRCC. There are differences in the distribution of body fat by sex, such that men are prone to have more visceral fat tissue, while women are prone to have more fat in the subcutaneous tissue [13]. Additionally, fat tissue secretes inflammatory cytokines that alter metabolic demands and increase the risk of cancer development [25]. Since women have a low tolerance for visceral fat storage, this may cause an increased risk of cancer in women. In this study, we found that VF% had the highest OR in the univariate and multivariate analyses.

Differences in the level of the sex hormone estrogen may provide an explanation for the association of VF% with increased risk of ccRCC. Estrogen can ease fat tissue deposition and is more abundant in women. It has alpha and beta receptors. It has been reported that the beta receptor reduces cell growth, migration, and invasion ability and increases apoptosis in RCC [13]. Additionally, an increased alpha to beta receptor ratio restricts visceral fat tissue deposition in premenopausal women [26]. In this study, we could not investigate the blood estrogen level.

This study had a number of limitations. First, the use of retrospective analysis could lead to selection bias. Second, the number of patients was also relatively small. Third, the CT images were evaluated based on a consensus, and we did not evaluate the inter- or intraobserver variability in this study. Fourth, there were no laboratory parameters, such as blood estrogen data and lipid panels. Finally, the adipose tissue area was evaluated from a single CT section instead of via a volume calculation.

In conclusion, significant differences were found in VFA and VF% in high-grade ccRCC in female patients unlike in male patients in this study. The sex-specific visceral fat composition represented by VFA and VF% could be used for estimating WHO/ISUP nuclear grade in patients with ccRCC. However, extensive studies with larger populations are needed to clearly confirm the relationships between sex-specific differences in visceral obesity and ccRCC.

## Figures and Tables

**Table 1:** The baseline characteristics and visceral fat measurements of patients.

	Total	Low-grade (n = 58)	High grade (n = 37)	p-value
Age (years)	59.6 ± 11.7 (34–86)	59.1 ± 11.4 (35–86)	60.3 ± 12.2 (34–86)	0.606
Sex (M/F)	56/39	39/19	17/20	0.040
Height (cm)	159.8 ± 7.7 (143–180)	159.4 ± 8.5 (143–180)	160.4 ± 6.2 (143–170)	0.534
Weight (kg)	76.2 ± 11.8 (52–114)	76.3 ± 13.4 (52–114)	76.0 ± 8.8 (60–93)	0.902
BMI (kg/m^2^)	29.3 ± 6.1 (18.9–46.6)	29.9 ± 6.5 (18.9–46.4)	28.2 ± 5.3 (19.7–46.6)	0.186
MTD (mm)	61.8 ± 36.8 (16–194)	49.1 ± 34.2 (16–112)	82.4 ± 44.0 (22–194)	<0.001
VFA (cm^2^)	152.0 ± 69.8 (12.8–296.7)	139.5 ± 71.6 (12.8–296.7)	171.6 ± 62.9 (15.0–272.8)	0.002
SFA (cm^2^)	223.8 ± 112.7 (18.4–606.1)	216.0 ± 112.6 (29.8–606.1)	236.1± 113.1 (18.4–552.5)	0.321
TFA (cm^2^)	378.2 ± 154.1 (33.4–863.4)	359.3± 154.1 (54.9–863.4)	407.8 ± 151.5 (33.4–739.2)	0.087
VF%	40.8 ± 12.0 (15.8–76.3)	38.8 ± 12.1 (15.8–76.3)	43.9 ± 11.2 (25.2–71.2)	0.037

BMI: body mass index; MTD: maximal tumor diameter; VFA: visceral fat area; SFA: subcutaneous fat area; TFA: total fat area; VF%: percentage of visceral fat.

**Table 2:** The characteristics of the low-grade and high-grade groups based on sex.

	Male (n = 56)	Female (n = 39)	p-value	Male			Female		
				Low-grade (n = 39)	High-grade (n = 17)	p-value	Low-grade (n = 19)	High-grade (n = 20)	p value
Age (years)	59.5 ± 11.1	59.7 ± 12.6	0.925	58.7 ± 11.0	61.3 ± 11.4	0.441	60.0 ± 12.4	59.5 ± 13.1	0.894
Height (cm)	160.7 ± 6.8	158.5 ± 8.7	0.174	159.5 ± 6.2	163.3 ± 7.6	0.056	159.1 ± 12.1	157.9 ± 5.8	0.687
Weight (kg)	76.0 ± 11.6	76.5 ± 12.2	0.840	75.0 ± 12.8	78.2 ± 8.0	0.354	79.0 ± 14.3	74.2 ± 12.6	0.227
BMI (kg/m^2^)	27.9 ± 5.6	31.2 ± 6.2	0.010	28.7 ± 6.1	26.2 ± 4.6	0.141	32.5 ± 6.5	29.9 ± 5.8	0.199
MTD (mm)	64.3 ± 38.0	58.4 ± 35.2	0.450	52.1 ± 26.2	93.9 ± 46.2	<0.001	42.8 ± 18.4	73.9 ± 41.1	0.006
VFA (cm^2^)	148.5 ± 74.4	157.1 ± 63.3	0.558	144.8 ± 73.6	156.9 ± 77.7	0.582	128.5 ± 68.0	184.2 ± 45.2	0.005
SFA (cm^2^)	187.0 ± 85.8	276.7 ± 126.0	<0.001	180.7 ± 75.2	201.4 ± 107.3	0.412	288.3± 141.6	265.6 ± 111.7	0.581
TFA (cm^2^)	339.5± 137.8	433.8± 161.1	0.003	331.4± 124.9	358.3± 166.3	0.505	416.8 ± 191.4	349.9± 127.1	0.529
VF%	43.3 ± 11.0	37.2 ± 12.6	0.014	42.3 ± 10.9	45.4 ± 12.3	0.338	31.4 ± 11.5	42.6 ± 11.3	0.004

BMI: body mass index; MTD: maximal tumor diameter; VFA: visceral fat area; SFA: subcutaneous fat area; TFA: total fat area; VF%: percentage of visceral fat.

**Table 3:** Results of receiver operating characteristic analysis.

	Total			Sex					
				Male (n = 56)			Female (n = 39)		
	VFA	VF%	MTD	VFA	VF%	MTD	VFA	VF%	MTD
AUC	0.643	0.627	0.735	0.552	0.588	0.780	0.753	0.787	0.733
Cutoff level	154.8	40.7	54.0	157.7	42.7	63.0	164.7	36.2	51.0
Sensitivity (%)	67.5	67.5	69.4	58.8	64.7	68.7	70.0	70.0	65.0
Specificity (%)	55.1	58.6	65.5	61.4	53.8	76.9	73.6	73.7	78.9
PPV(%)	49.0	51.0	55.6	40.0	37.9	55.0	73.7	73.7	76.5
NPV(%)	72.7	73.9	77.6	77.4	77.8	85.7	70.0	70.0	68.2

AUC: area under the curve; MTD: maximal tumor diameter; VFA: visceral fat area; VF%: percentage of visceral fat; PPV: positive predictive value; NPV: negative predictive value.

**Table 4:** Univariate analysis for predicting high-grade ccRCC.

	Total (n = 95)			Male (n = 56)			Female (n = 39)		
	OR	%95 CI	p-value	OR	%95 CI	p-value	OR	%95 CI	p-value
Age (years)	1.00	0.97–1.04	0.643	1.02	0.96–1.07	0.429	0.99	0.95–1.05	0.890
MTD (mm)	1.03	1.01–1.04	<0.001	1.03	1.01–1.06	<0.001	1.03	1.00–1.07	0.002
VFA, cm^2^	1.00	1.00–1.01	0.026	1.00	0.99–1.01	0.574	1.01	1.00–1.04	0.003
SFA, cm^2^	1.00	0.99–1.00	0.393	1.00	0.99–1.01	0.402	0.99	0.98–1.00	0.568
TFA, cm^2^	1.00	0.99–1.00	0.131	1.00	0.99–1.01	0.494	1.00	0.99–1.00	0.514
VF%	1.03	1.00–1.07	0.039	1.02	0.97–10.7	0.332	1.09	1.02–1.17	0.002

MTD: maximal tumor diameter; VFA: visceral fat area; SFA: subcutaneous fat area; TFA: total fat area; VF%: percentage of visceral fat.

**Table 5:** Multivariate analysis for predicting high-grade ccRCC.

	Total (n = 95)			Male (n = 56)			Female (n = 39)		
	OR	%95 CI	p-value	OR	%95 CI	p-value	OR	%95 CI	p-value
MTD (mm)	0.95	0.94–0.97	<0.001	0.95	0.93–0.98	0.001	0.93	0.88–0.98	0.011
VFA, cm^2^	0.98	0.97–0.99	0.004	0.99	0.97–1.00	0.142	0.97	0.95–0.99	0.022
VF%	1.00	0.95–1.06	0.765	1.00	0.92–1.08	0.970	0.95	0.87–1.04	0.353

MTD: maximal tumor diameter; VFA: visceral fat area; VF%: percentage of visceral f
